# Supplementary material for: Identification of photoperiod-induced specific miRNAs in the adrenal glands of Sunite sheep (Ovis aries)
Source: Front Vet Sci. 2022 Jul 22;9:888207. doi: 10.3389/fvets.2022.888207 (PMC9354845; doi:10.3389/fvets.2022.888207)
Supplement: Supplementary file 1 [file Data_Sheet_1.ZIP › Supplementary file/miRDeep2 file.zip!/miRDeep2 file/Novel_Structure/SP42A2/Novel_480.pdf]

Provisional ID	:	NC_019460.2_11236
Score total	:	31.8
Score for star read(s)	:	3.9
Score for read counts	:	25.1
Score for mfe	:	1.2
Score for randfold	:	1.6
Score for cons. seed	:	
Total read count	:	61
Mature read count	:	57
Loop read count	:	0
Star read count	:	4

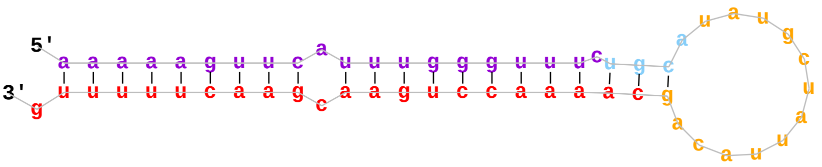

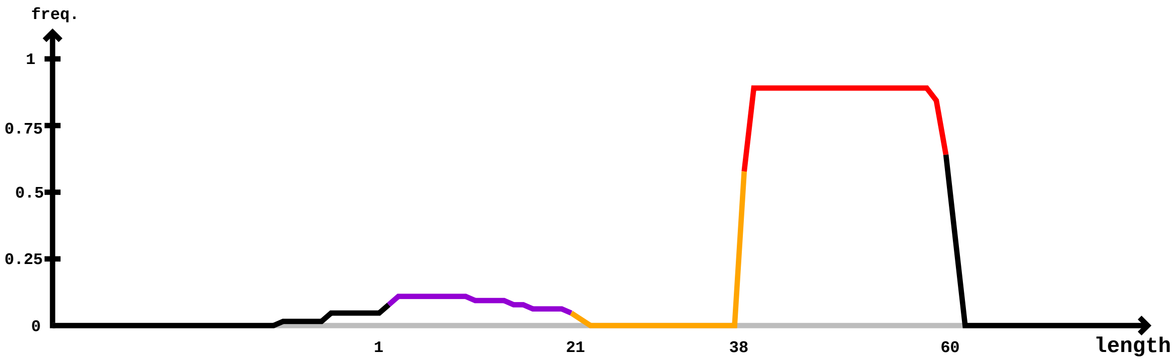


```
                                     Star                                  Mature
5'- aauuccacaagcaaugauugaauugguuuggccaaaaaguucauuuggguuucugcauaugcuauuacagcaaaaccugaacgaacuuuuuguccaacccaauagaugcaaa   -3'    obs
    aauuccacaagcaaugauugaauugguuuggccaaaaaguucauuuggguuucugcauaugcuauuacagcaaaaccugaacgaacuuuuuguccaacccaauagaugcaaa          exp
    ..............((..(..(((((.((((.((((((((((.(((((((((.(((.............))))))))))))).))))))))))).)))).))))).)..))..   reads      mm        sample
    ......................uuggAuuggccaaaaaguuc......................................................................   1          1         seq
    ...........................uuggccaaaaagCucauuu..................................................................   1          1         seq
    ...........................uuggccGaaaaguucauuugg................................................................   1          1         seq
    .................................aaaaaguucGuuuggguuuc...........................................................   2          1         seq
    ..................................Uaaaguucauuuggguuu............................................................   1          1         seq
    ..................................aaaaguucGuuuggguuucu..........................................................   1          1         seq
    ......................................................................caaaaccugaacgaacuuuuu.....................   2          0         seq
    ......................................................................Aaaaaccugaacgaacuuuuug....................   33         1         seq
    ......................................................................caaaaccugaacgaacuuuuug....................   2          0         seq
    .......................................................................aaaaUcugaacgaacuuuu......................   2          1         seq
    .......................................................................aaaaccugaacUaacuuuu......................   1          1         seq
    .......................................................................aaaaccAgaacgaacuuuuu.....................   11         1         seq
    .......................................................................aaaaccugaacUaacuuuuug....................   4          1         seq
    .......................................................................aaaaccugaaGgaacuuuuug....................   1          1         seq
    .......................................................................aaaaccugaacgaacuuuuugC...................   1          1         seq
```
